# Multiplexed DNA Sequence Capture of Mitochondrial Genomes Using PCR Products

**DOI:** 10.1371/journal.pone.0014004

**Published:** 2010-11-16

**Authors:** Tomislav Maricic, Mark Whitten, Svante Pääbo

**Affiliations:** Department of Evolutionary Genetics, Max Planck Institute for Evolutionary Anthropology, Leipzig, Germany; Smithsonian Institution National Zoological Park, United States of America

## Abstract

**Background:**

To utilize the power of high-throughput sequencers, target enrichment methods have been developed. The majority of these require reagents and equipment that are only available from commercial vendors and are not suitable for the targets that are a few kilobases in length.

**Methodology/Principal Findings:**

We describe a novel and economical method in which custom made long-range PCR products are used to capture complete human mitochondrial genomes from complex DNA mixtures. We use the method to capture 46 complete mitochondrial genomes in parallel and we sequence them on a single lane of an Illumina GA_II_ instrument.

**Conclusions/Significance:**

This method is economical and simple and particularly suitable for targets that can be amplified by PCR and do not contain highly repetitive sequences such as mtDNA. It has applications in population genetics and forensics, as well as studies of ancient DNA.

## Introduction

Methods that enrich DNA samples for particular DNA sequences are important in order to allow efficient sequencing of targets that are present in complex mixtures of irrelevant DNA sequences. These may either be entire genomes of organisms under study or DNA from several organisms in environmental or medical samples [1], [2]. Methods that are able to “capture” relevant DNA sequences rely on hybridization of target sequences to probes that can be either in solution or immobilized on a surface (e.g. [3], [4], [5]). The hybridization is sometimes followed by extensions [2] or extension in combination with circularization of the probes [6]. Other methods rely on micro-droplet-based selection [7]. Although all these methods achieve their goals, they involve probes and/or equipment that have to be purchased from manufacturers at substantial costs as well as loss of time.

Here, we present a method where PCR products are used to capture targets for sequencing from pooled sequencing libraries of multiple individuals, using standard laboratory equipment. We apply this method to DNA pools of libraries from several human individuals from which we capture complete mitochondrial (mt) DNAs, a maternally inherited DNA molecule which is extensively studied in population genetics, medicine, forensics, and phylogenetics [8].

## Materials and Methods

### Production of indexed libraries

DNA extracts of 46 individuals from which the hypervariable region I had been sequenced [9] were used for indexed Solexa library preparation. First, eight hundred ng of DNA were sonicated (Bioruptor, Diogenode, Liege, Belgium) five times for seven minutes with the output selector switched to (H)igh. This fragmented the DNA to a range of 150 to 800 base-pairs. Two hundred ng were then used for the production of the indexed libraries as published [10], starting from the blunting step. In the last step of the protocol, the indexing amplification was run into plateau (20 cycles) and the reactions were purified using a MinElute PCR purification kit (Qiagen, Hilden, Germany). DNA concentrations of individual libraries were measured with a spectrophotometer (NanoDrop ND-1000, Thermo Scientific, Wilmington, DE, USA) and the libraries were pooled in equimolar amounts to a total of 2 µg.

### Bait production

Two overlapping long-range PCR products encompassing the whole mitochondrial genome were produced as described [11]; DNA extracted from the saliva of one individual was used as the template. The PCR products were purified using carboxyl-coated magnetic beads (SPRI beads, Agencourt AMPure XP, Agencourt, Beverly, MA, USA) and the concentration was measured by NanoDrop. The two products were pooled in equimolar amounts to a total amount of 3 µg; the pooled products were sonicated (Bioruptor) two times for seven minutes with the output selector switched to (H)igh which produced fragments from 150 to 850 bases long. The products were biotinylated by ligating the Bio-T/B adapter (sequence in Supplementary protocol S1), MinElute column purified, made single-stranded and immobilized on streptavidin-coated magnetic beads.

### Hybridization

The pooled libraries were made single-stranded and added to the bait-coated beads; the mixture was attached to a rotator and rotated at 65°C in a hybridization oven (SciGene, Model 700, Sunnyvale, CA, USA). After 48 hours, library molecules that did not hybridize to the bait were washed away and the enriched library pool was eluted by heating for 3 minutes at 95°C. The DNA concentration was measured by qPCR (Mx3005P Real Time PCR System, Stratagene, La Jolla, CA), the pool was further amplified for 15 cycles using the bridge primers (sequence in Supplementary protocol S1) and purified with the SPRI beads; the concentration of the 22 µl eluate was determined with the Bioanalyzer 2100 DNA 1000 chip (Agilent Santa Clara, CA).

### Sequencing

Libraries were sequenced with 76+7 cycles on one lane of an Illumina flow cell (Cluster Generation kit V2, FC-103-300x sequencing chemistry) according to the manufacturer's instructions for Single Read Multiplex sequencing on the Genome Analyzer IIx platform. The run was processed with RTA 1.5 (Illumina Inc.). Afterwards, the PhiX 174 control reads were aligned to the corresponding reference sequence to obtain a training data set for the base caller Ibis [12]. Raw sequences called from Ibis were separated by sample using their index read (allowing one mismatch and the loss of the first base) [12]. Sequences obtained for each sample were searched for the adapter sequence (AGATCGGAAGAGCACACGTCTGAACTCCAG) and read ends trimmed back when they could represent adapter sequence. Further, reads were filtered for sequence quality and complexity. In this step, reads having more than 5 bases with a quality score below 10 (PHRED score) [12] and reads with sequence entropy below 0.85 were removed (where entropy was calculated by summing -p*log2(p) for each of the four bases; p is the frequency of a base in the read).

### Assembly

The reads for each of the 46 samples were mapped to the revised Cambridge reference mitochondrial sequence (NC012920.1) using the iterative mapping assembler MIA [13]. Mapping allowed for up to four mismatches or three mismatches and one indel in a 76 base long read. Reads starting and ending at the same coordinate were then collapsed, making one consensus read by taking the highest quality base for each position [14]. From the mapped reads, consensus mitochondrial sequence was called: a base was called in the consensus sequence if the score for the base was a positive number (200 points are given for match, −600 for mismatch, and −100 for an N in the read), otherwise an N was called.

## Results and Discussion

**Figure 1:**
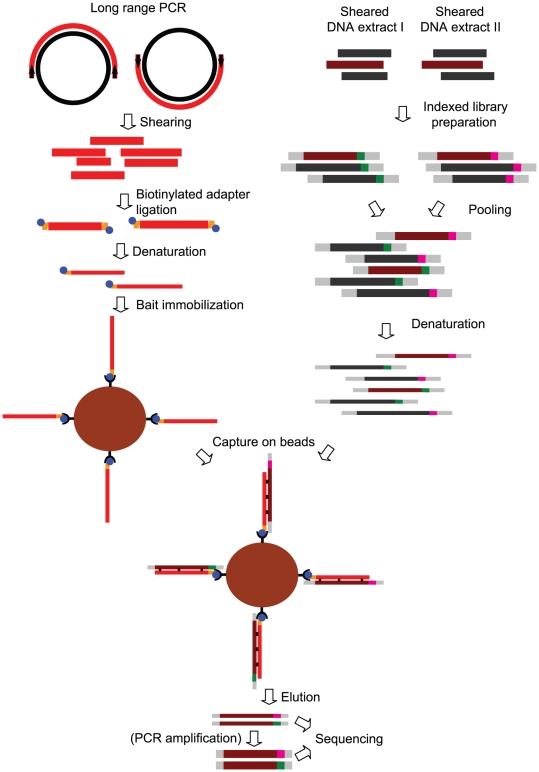
Overview of the capture-on-beads method. On the left the production of the immobilized bait from two long range PCR products is shown; on the right the production of a pool of indexed libraries which are used in the capture (bottom). The eluted molecules can either be sequenced directly or first amplified and then sequenced. The bait is light red, mitochondrial DNA in the libraries is dark red, indices are shown in green and pink, adapters in gray. Thicker lines represent double stranded DNA while thinner lines represent single stranded DNA.

Equimolar amounts of two long-range PCR products which together encompass the complete mitochondrial genome, which is a double-stranded circular molecule of 16,6 kb, were pooled and fragmented by sonication, ligated to a biotinylated DNA adapter, denatured, and immobilized on streptavidin-coated magnetic beads (Figure 1, top left). The immobilization prevents self-hybridization of the bait molecules that occur if they are free in solution. DNA extracted from blood or saliva from 46 individuals [9] were used to produce indexed Solexa libraries [10], which were pooled in equimolar amounts, denatured (Figure 1, top right) and incubated with the beads for 48 hours. The beads were then washed and the captured molecules were heat-eluted, amplified and sequenced (Figure 1, bottom) on one lane of a Solexa Genome Analyzer II.

**Figure 2:**
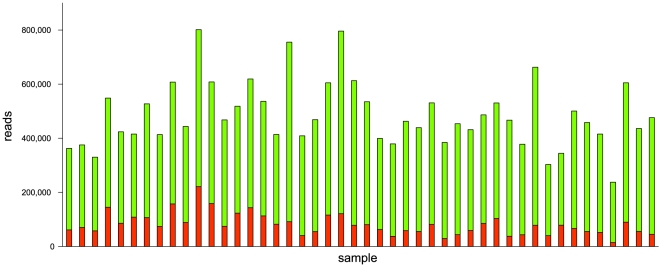
Number of reads sequenced (green bar) and aligned to the mitochondrial genome (red bar) for each sample.

**Figure 3:**
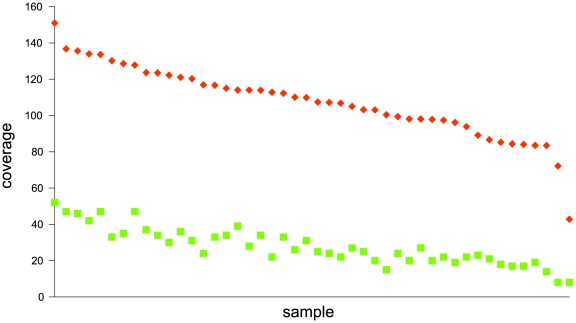
Average (red squares) and minimum coverage (green squares) of the mitochondrial genome for each sample.

**Figure 4:**
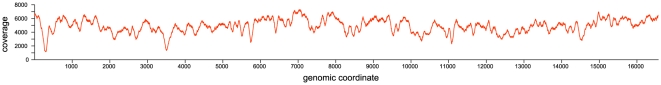
Coverage of each position across the whole mitochondrial genome, considering all the samples together.

The number of reads per individual varied between 237,763 and 801,556 (Figure 2). On average, 16% of the reads in each sample mapped [14] to the reference mtDNA sequence (NC_012920) (Figure 2) and the average mtDNA coverage varied between 43- and 151-fold (Figure 3). The minimum coverage at any base in any sample was 8-fold (Figure 3). The coverage across the mitochondrial genome and samples was fairly uniform, with a 6-fold difference between the positions of highest and lowest coverage (Figure 4).

To validate the method, we compared the sequences determined by us to sequences for parts (hypervariable region I) of the same mtDNAs produced by a traditional approach where PCR products were sequenced by the Sanger method [9]. After the exclusion of a homopolymeric C-stretch which can vary in length due to PCR-induced nucleotide misincorporations, a total of 17,134 bases (approximately 372 per individual) could be compared. They agreed except at seven positions in single individuals, where Ns were called by the capture/Solexa method. These Ns most probably arise due to rare recombination events during the amplification of the pool of indexed libraries and can be avoided by omitting this step [10], [15]. One N was called both in the PCR/Sanger and the capture/Solexa in one individual. This is probably due to heteroplasmy, i.e. the presence of two different mtDNA sequences in this individual.

*Numts* are insertions of parts of mitochondrial genome into the nuclear genome [8]. Because of their similarity to the mitochondrial genome *numts* can potentially hybridize to the mitochondrial DNA-derived baits and lead to ambiguities in mtDNA sequences (represented as Ns) or even to incorrect sequence determination. To test for the potential presence of *numts* we mapped all the reads overlapping ambiguous positions (Ns) against the human genome with blat [16]. Only 0.08% of the reads had a higher score to the nuclear genome then to the organellar mtDNA and are thus potentially *numts*. Additionally, we translated all protein-coding sequences *in silico* (13 per mitochondrial sequence) and found no premature stop codons. This demonstrates that the capture method is reasonably insensitive to human *numts*.

The method described allows the efficient capture of any unique sequence for which a PCR product can be generated. It is cost efficient in that it requires only standard laboratory equipment and reagents and fast in that the capture can be performed immediately when the PCR products are at hand. A similar method for capturing mtDNAs was recently described [5]. The authors performed 100 PCR reactions to produce biotinylated baits covering the mtDNA and performed two consecutive hybridizations in solution. The approach presented here is different in that the bait is immobilized on the beads during capture. This prevents the bait molecules from self-hybridizing making both strands accessible for the target capture and the production of the bait simpler (e.g. only two PCR reactions are needed). Additionally, we have shown that our approach can be multiplexed, allowing for efficient analysis of many samples in parallel. In our research group it has been used to capture complete mitochondrial genomes from complex samples such as saliva and ancient hominin bones. Although the efficiency of capture is slightly lower when the human DNA is contaminated by one or two orders of magnitude greater amounts of microbial DNA, it is possible to retrieve complete mitochondrial genomes from most such samples using this method.

## Supporting Information

Protocol S1 Detailed protocol of the capture-on-beads method.
(0.12 MB DOC)
