# Supplementary material for: Multiplexed DNA Sequence Capture of Mitochondrial Genomes Using PCR Products
Source: PLoS One. 2010 Nov 16;5(11):e14004. doi: 10.1371/journal.pone.0014004 (PMC2982832; doi:10.1371/journal.pone.0014004)
Supplement: Protocol S1 — Detailed protocol of the capture-on-beads method. (0.12 MB DOC) [file pone.0014004.s001.doc]

Protocol For:

**Multiplexed DNA sequence capture of mitochondrial genomes using PCR products**

**Tomislav Maricic, Mark Whitten and Svante Pääbo**

*Department of Evolutionary Genetics, Max Planck Institute for Evolutionary Anthropology, Leipzig, Germany*

**LEGEND**

🢡***ATTENTION***

* ***HINT***

**🖐*REST***

**REAGENTS**

M-270 streptavidin beads (Dynal, Oslo, Norway)

Quick blunting kit (NEB, Ipswich, MA, USA)

Quick ligation kit (NEB)

Agilent aCGH Hybridization Kit 5188-5220 (Agilent, Santa Clara, CA)

- Agilent blocking agent 10X
- Agilent hybridization buffer 2X

NaOH (10M)

Acetic acid (20%)

Taq Gold Polymerase (Applied Biosystems, Foster City, CA, USA)

Phusion HF DNA Polymerase (Finnzymes Oy, Espoo, Finland)

Tween-20

Oligos (written in 5’ to 3’ direction):

Bio-T (1,000 µM): Biotin-TCAAGGACATCC*G

B (1,000 µM): CGGATGTCCTT*G

BO1.P5.F (200 µM): AATGATACGGCGACCACCGAGATCTACACTCTTTCCCTACACGACGCTCTTCCGATCT-phosphate

BO2.P5.R (200 µM): AGATCGGAAGAGCGTCGTGTAGGGAAAGAGTGTAGATCTCGGTGGTCGCCGTATCATT-phosphate

BO3.P7.part1.F (200 µM): AGATCGGAAGAGCACACGTCTGAACTCCAGTCAC-phosphate

BO4.P7.part1.R (200 µM): GTGACTGGAGTTCAGACGTGTGCTCTTCCGATCT-phosphate

BO5.P7.part2.F (200 µM): ATCTCGTATGCCGTCTTCTGCTTG-phosphate

BO6.P7.part2.R (200 µM): CAAGCAGAAGACGGCATACGAGAT-phosphate

Sol_bridge_P5 (10 µM): AATGATACGGCGACCACCGA

Sol_bridge_P7 (10 µM): CAAGCAGAAGACGGCATACGA

* optional phosphorothioate bond to render the oligos resistant to nuclease degradation

**PROCEDURE**

PREPARATION OF THE BAIT

**Preparation of the bait for one capture experiment is described here.*

1. Do two or three long range PCRs that encompass the complete mitochondrial genome as in e.g. [1]. Check for successful amplification by running the products on an agarose gel. Purify the products and measure their concentration with a spectrophotometer.
2. Pool the purified long-range PCR products in equimolar amounts; for one reaction 1.5 µg of DNA in total are needed.
3. Shear the DNA fragments so they have an average fragment size below one kilobase; if the Bioruptor sonication system is used, dilute the DNA to a total volume of 80ul and sonicate 2 times for 6 minutes with the output selector switched to (H)igh.
4. Run ~200ng of the sonicated DNA on a 2% agarose gel to check if the sonication was successful (the smear should be brightest at a size lower than one kilobase and no fragment longer than five kilobases should be visible)

**🖐 *Sheared bait DNA can be stored at -20 °C for many months in a siliconized tube***

1. Blunt ending (NEB Quick Blunting kit):
   - Sheared DNA (1.3 µg) 76 µl
   - Blunting buffer (10X) 10 µl
   - dNTP mix (1mM) 10 µl
   - Blunting Enzyme Mix 4 µl
   - (total 100 µl)

Incubate the reaction at RT for 30 minutes.

6. Purify the blunt-ended DNA using one MinElute column and the standard protocol and elute it in 15 µl of EBT buffer

**If higher amounts of DNA are used for blunt ending, one can use QiaQuick columns that have a higher DNA binding capacity. Maximum binding capacity of the MinElute columns is 5 µg of DNA, and of QiaQuick columns is 10 µg; minimum elution volumes are 15 and 30 µl, respectively. When the minimum elution volume is used, the elution buffer should be left on the silica membrane for one minute before centrifuging the columns to ensure maximum recovery****.***

**🖐 *Purified bait can be stored at -20 °C for many months in a siliconized tube***

1. Adapter ligation (NEB Quick ligation kit):

- Blunt-ended DNA 15 µl
- Adapters Bio-T/B (50uM) 1 µl
- Quick ligase buffer (2X) 20 µl
- Quick ligase 4 µl
- (total 40 µl)

Incubate the reaction at RT for 15 minutes

🢡*Mix the DNA with the adapters before adding the ligase to prevent extensive chimera formation.*

1. MinElute purify the reaction and elute in 15 µl of EBT buffer.
2. Measure the concentration of DNA on a spectrophotometer; expected total mass of DNA is more than 500 ng

**🖐*Purified bait (dsDNA) can be stored at -20 °C for many months in a siliconized tube***

PREPARATION OF THE INDEXED LIBRARIES

Two micrograms of the indexed libraries are required for one capture. The capture of both ancient and modern DNA libraries is possible; the sizes of the inserts can range from 30 – 1000 bases; a mean size of ~500 is recommended.

1. Prepare the indexed libraries as described in [2]
2. Pool the purified libraries in equimolar amounts to total of 2 µg.

🢡*If there is less than 2 µg of DNA then amplify the libraries before pooling them. Amplifying the pool is not recommended since recombinant molecules (molecules containing an index from one sample and insert from another sample) can be created. Amplification of modern DNA libraries can be done with a proofreading polymerase such as Phusion HF DNA polymerase. Amplification of ancient DNA libraries can be done with the Taq Gold polymerase that can read over the uracil-containing nucleotides.*

ADDING THE BAIT TO THE BEADS

**Streptavidin may come off the beads more easily if it is connected to a DNA molecule which is why we recommended always making fresh bait-containing beads.*

Maximally 20-25 ng of the bait DNA can bind to 1 µl of the M-270 beads. However, since the combined efficiency of the adapter ligation and binding to the beads is ~30 % (in other words, maximally ~30% of the adapter-ligated bait will bind to the theoretically endless amount of the beads) 400-500 ng of the Bio-T/B adapter-ligated bait DNA is required per capture to saturate 5 µl of the M-270 beads.

1. Combine 500 ng of the Bio-T/B adapter-ligated bait DNA in a volume of 5-50 µl with the same volume of BWT buffer (2X)
2. Heat the mixture for 1 min at 98 °C, put on ice immediately and keep cold until further use
3. Wash 5 µl of the M-270 beads with 200 µl of BWT buffer (1X) (*washing the beads* means: resuspending the beads in the buffer, collecting the beads with a magnet on the tube wall and removing the supernatant)
4. Wash the beads with 200 µl TET buffer
5. Add the bait mixture that is kept on ice to the beads and resuspend the beads
6. Rotate the tube for 20 minutes at RT (or shake the tube occasionally) to allow the biotinylated bait to bind to the streptavidin beads. Ensure that the lids are closed
7. Collect the beads with a magnet on the tube wall and remove the supernatant which contains the bait that did not bind to the beads
8. Wash the beads 2 times with 200 µl of the BWT buffer (1X) heated to 50ºC
9. Resuspend the beads in 50 µl of TET buffer, transfer the suspension to a new 0.2 µl PCR tube and store it at 4 ºC until the hybridization mixture is ready

STARTING THE CAPTURE

1. Heat the hybridization oven to 65ºC
2. Prepare the hybridization mixture:

- 2 µg of the pooled indexed libraries 17.8 µl
- BO1.P5.F (200 µM) 0.5 µl
- BO2.P5.R (200 µM) 0.5 µl
- BO3.P7.part1.F (200 µM) 0.5 µl
- BO4.P7.part1.R (200 µM) 0.5 µl
- BO5.P7.part2.F (200 µM) 0.5 µl
- BO6.P7.part2.R (200 µM) 0.5 µl
- Agilent blocking agent 10X 5.2 µl
- Agilent hybridization buffer 2X 26 µl
- total (52 µl)

🢡*If the indexed libraries pool is in a volume that is higher than 17.8 µl, but not higher than 70 µl, then increase the other mixture components proportionally (the amount of beads need not be increased). If the library pool is in a volume higher than 70 µl, then concentrate it, e.g. by MinElute.*

**The six blocking oligos (BO1-6) are the same as in [2]. They hybridize to the Solexa adapters except to the index sequence. They are used to prevent cross-linking of library adapters which could lead to unwanted capture of non-targeted library molecules. The blockers are phosphorylated at their 3’ ends to prevent them from serving as PCR primers in further PCR amplifications of the enriched library.*

1. Incubate the mixture for 3 minutes at 95ºC to denature the DNA
2. Transfer the tube to a heat block and incubate for 30 minutes at 37 ºC
3. Spin down briefly
4. Remove the TET buffer from bait-coated beads from step 20 by magnetically collecting them and discarding the supernatant
5. Add 52 µl of the mixture to the ‘dried’ beads and resuspend the beads

🢡*Sometimes the beads cannot be resuspended immediately but they will resuspend during the later incubation at 65ºC.*

1. Use tape to secure the lid
2. Use tape to attach the tubes to the rotor inside the hybridization oven and rotate slowly (12 rpm)

**Alternatively, if there are no hybridization ovens in the lab, one can incubate the mixture for two nights at 65* *ºC in a slowly shaking heating block.*

1. Incubate the mixture in the oven at 65ºC for two nights

**It is important that the tube used is tightly sealed.*

1. Check after one hour if the taped lid is holding and there is no leakage

BEADS WASHING AND ENRICHED LIBRARY ELUTION

1. Take the tube out of the hybridization oven/heating block
2. Collect the beads with a magnet on the tube wall and discard the supernatant
3. Wash the beads 3 times with 200 µl BWT buffer (1X)
4. Add 200 µl HWT buffer, which has been preheated to 60ºC, to the beads; incubate at 60ºC for 2 minutes on a heat block; collect the beads with a magnet and discard the supernatant; repeat once
5. Wash the beads with 200 µl BWT buffer (1X)
6. Add 100 µl TET buffer and transfer the suspension to a new tube
7. Collect the beads with a magnet on the tube wall and discard the supernatant
8. Resuspend the beads in 50 µl NaOH (125 mM)
9. Vortex for 10 sec
10. Magnetically collect the beads and transfer the supernatant to a neutralizing solution (500 µl PBI buffer from the MinElute kit and 3.8 µl of 20% acetic acid)

🢡*Here the supernatant is not discarded, instead it is kept since it contains the enriched library!*

1. Repeat from step 39, adding the supernatant into the same neutralizing solution from the last step. In case the pH indicator in MinElute’s PBI buffer is violet, add more acetic acid until it turns yellow.

**Keep the beads in case the NaOH melting did not work.*

1. MinElute purify the mixture from the previous step; elute the DNA in 15 µl of TET or EBT. Keep the enriched library pool in a siliconized tube at -20 ºC.

ENRICHED POOL QUANTIFICATION

1. Quantify the enriched library using qPCR as described in [3] using the primers Sol_bridge_P5 and Sol_bridge_P7 (the library is expected to contain more than 10^9^ molecules in total). The pool of enriched indexed libraries is now ready for direct sequencing.

🢡*If possible, any amplification of the pooled indexed libraries should be avoided, since recombinant DNA molecules could be created [4]. For sequencing of the library on one lane of the Genome analyzer II at least 3-7*10^8^ of library molecules are required in a volume of not more than 10 µl (higher volumes are probably possible but have not been tested). In the next step the library can be mixed with the hybridization buffer by a sequencing technician to a total volume of 120 µl and put into the cluster generation step of the Genome Analyzer II protocol*

**RECIPES**

TET buffer

10 mM Tris-Cl, pH 8.0

1 mM EDTA, pH 8.0

0.05% Tween-20

EBT buffer

EB buffer from MinElute kit

0.05% Tween-20

Oligo hybridization buffer (10x)

500 mM NaCl

10 nM Tris-Cl, pH 8.0

1 nM EDTA, pH 8.0

BWT buffer (2X)

2 M NaCl

10 mM Tris-Cl, pH 8.0

1 mM EDTA, pH 8.0

0.1% Tween-20

BWT buffer (1X)

BWT buffer (2X) diluted 2 times in water

HWT buffer

2.5mM MgCl

50 mM KCl

15 mM Tris-HCl (pH 8.0)

0.1% Tween-20

(KCl and Tris-HCl together make the GeneAmp PCR Gold Buffer(Applied Biosystems))

NaOH 125 mM

Always make fresh from a 10M NaOH stock.

Adapters Bio-T/B (50 µM)

Oligo Bio-T 12 µl

Oligo B 12 µl

10X Oligo hybridization buffer 3 µl

Water 3 µl

Heat the mix to 95 ºC for 5 seconds, then ramp to 15 ºC at the rate of -0.1 ºC/sec. Dilute the mix in 210 µl of water.

**TROUBLESHOOTING**

AFTER DNA SHEARING THERE ARE STILL FRAGMENTS OF SEVERAL KILOBASES VISIBLE ON THE GEL

If the Bioruptor sonicator was used, it is possible that the DNA solution droplets have collected on the tube walls and tube cap and were therefore not sonicated. Do shorter cycles and spin down the tube between each cycle.

THE AMOUNT OF MOLECULES IN THE FINAL ELUATE IS TOO LOW

A possible reason for the low amount of the library molecules is that the NaOH melting did not work properly [5]. Resuspend the beads in 20 µl of TET buffer, heat to 95 ºC and then collect the supernatant after collecting the beads with a magnet on the tube wall. NaOH melting is preferred over heat melting since in the NaOH melting only the captured library ends up in the final eluate. When using heat melting, the bait DNA is eluted together with the library. The presence of the bait DNA should in theory not pose problems for further sequencing or amplification, but should be avoided if possible.

Another possible reason for the low amount of the library molecules is that the bait was not attached to the beads due to failure of DNA blunting, adapter ligation or binding to the beads. Use a PCR product as a positive control to find out if there were problems in any of the steps.

**EQUIPMENT**

DNA fragmentation device (e.g. Bioruptor sonicator (Diogenode, Liege, Belgium))

MinElute columns (Qiagen, Hilden, Germany)

Equipment and reagents for indexed libraries preparation [2]

PCR cycler

Magnetic particle concentrator (e.g. Invitrogen, Karlsruhe, Germany, cat. no 120-20D)

Spectrophotometer (e.g. NanoDrop ND-1000 (NanoDrop Technologies, Wilmington, USA))

Hybridization oven (SciGEne, Model 700, Sunnyvale, CA, USA) or heating block (e.g.

Eppendorf Thermomixer comfort)

Laboratory tape (Roth, Karlsruhe, Germany)

qPCR machine (e.g. Mx 3005P Real Time PCR System, Stratagene, La Jolla, CA)

Siliconized polypropylene microcentrifuge tubes (Sigma, Sigma Aldrich, St. Louis, MO, USA)

Microcentrifuge tubes (Eppendorf)

0.2 µl PCR tubes

Reference List

1. Meyer M, Stenzel U, Myles S, Prufer K, Hofreiter M (2007) Targeted high-throughput sequencing of tagged nucleic acid samples. Nucleic Acids Res 35: e97.

2. Meyer M, Kircher M (2010) Illumina sequencing library preparation for highly multiplexed target capture and sequencing. Cold Spring Harb Protoc 2010: pdb prot5448.

3. Meyer M, Briggs AW, Maricic T, Hober B, Hoffner B, et al. (2008) From micrograms to picograms: quantitative PCR reduces the material demands of high-throughput sequencing. Nucleic Acids Res 36: e5.

4. Meyerhans A, Vartanian JP, Wain-Hobson S (1990) DNA recombination during PCR. Nucleic Acids Res 18: 1687-1691.

5. Maricic T, Paabo S (2009) Optimization of 454 sequencing library preparation from small amounts of DNA permits sequence determination of both DNA strands. Biotechniques 46: 51-52, 54-57.
